# Current Update of Laboratory Molecular Diagnostics Advancement in Management of Colorectal Cancer (CRC)

**DOI:** 10.3390/diagnostics10010009

**Published:** 2019-12-23

**Authors:** Siew-Wai Pang, Noel Jacques Awi, Subasri Armon, Wendy Wan-Dee Lim, John Seng-Hooi Low, Kaik-Boo Peh, Suat-Cheng Peh, Sin-Yeang Teow

**Affiliations:** 1 Department of Medical Sciences, School of Healthcare and Medical Sciences, Sunway University, Jalan Universiti, Bandar Sunway, Subang Jaya 47500, Malaysia; 2 Pathology Department, Hospital Kuala Lumpur, Jalan Pahang, Kuala Lumpur 50588, Malaysia; 3 Sunway Medical Centre, Jalan Lagoon Selatan, Bandar Sunway, Subang Jaya 47500, Malaysia; 4 Mahkota Medical Centre, Mahkota Melaka, Jalan Merdeka, Melaka 75000, Malaysia

**Keywords:** colorectal cancer, molecular, diagnostics, biomarker, technologies, personalized medicine, therapy, genetic, screening, challenges

## Abstract

Colorectal cancer (CRC) continues to be one of the most common cancers globally. The incidence has increased in developing countries in the past few decades, this could be partly attributed to aging populations and unhealthy lifestyles. While the treatment of CRC has seen significant improvement since the advent of target-specific therapies and personalized medicine, CRC is oftentimes detected at late or advanced stages, thereby reducing the efficacy of treatment. Hence, screening for early detection is still the key to combat CRC and to increase overall survival (OS). Considering that the field of medical diagnostics is moving towards molecular diagnostics, CRC can now be effectively screened and diagnosed with high accuracy and sensitivity. Depending on the tumor genotype and genetic profile of the individual, personalized treatments including tyrosine kinase inhibitor therapy and immunotherapy can be administered. Notably, there can be no one single treatment that is effective for all CRC patients due to the variation in tumor genetics, which highlights the importance of molecular diagnostics. This review provides insights on therapeutic modalities, molecular biomarkers, advancement of diagnostic technologies, and current challenges in managing CRC.

## 1. Colorectal Cancer (CRC) and Its Treatment

**Figure 1:**
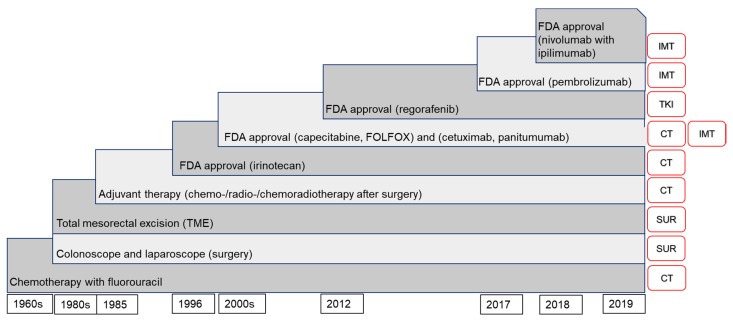
Simplified timeline of colorectal cancer (CRC) treatment options reviewed. The timeline is not constructed according to scale. The red boxes on the right represent the type of treatment, where CT = chemotherapy, SUR = surgery, IMT = immunotherapy, TKI = tyrosine kinase inhibitor.

Colorectal cancer (CRC) is one of the most common cancers worldwide, ranking third and second for men and women, respectively. In 2018, Global Cancer Observatory (GLOBOCAN) estimated more than 1.8 million new CRC cases worldwide [1]. There are various therapeutic modalities for CRC depending on the pathological characteristics of the tumor. Laparoscopic surgery is normally carried out for early-stage primary disease, open surgery tumor resection for cases with metastases, and adjuvant radiotherapy for nonresectable cases. Other CRC therapies are neoadjuvant and palliative chemotherapies [2,3], immunotherapy [4], and tyrosine kinase inhibitor (TKI) therapy [5]. The advent of checkpoint inhibitors made of monoclonal antibodies (mAbs) have been a breakthrough for the treatment of several cancers including CRC [6]. The safety profile and its efficacy in treating certain CRC patient populations have led to multiple clinical trials. Figure 1 summarizes the list of CRC treatments reviewed according to their date of application or approval by the Food and Drug Administration (FDA) [7,8,9,10,11,12,13,14,15,16,17]. Despite advances in medical and surgical treatment, the long-term survival rates have not improved much [18]. One of the ways in which CRC can be treated optimally is through the elucidation of individual genetic profiles for personalized therapies or precision medicine. Since CRC has several tumor subtypes with complex mutations and genetic variations, treatment decision supported by molecular testing results can dramatically improve the survival rate [19]. The details and specifications of the genes will be discussed in the next section. With advancement of DNA sequencing technologies such as next-generation sequencing (NGS), it is optimistic that precision medicine may be more accessible and affordable, thereby improving clinical outcomes. The sub-sections below will discuss the current treatment methods for CRC.

## 2. Molecular Biomarkers

Molecular biomarker is an umbrella term for all biomarkers that can be measured based on the biomarker’s molecular characteristic. The detection of biomarkers has been the backbone for screening and diagnostic aid in medical laboratories. They are routinely carried out using patient samples such as tissue biopsy, blood, urine, and other biological samples. One of the most common detection methods used in clinical laboratories is immunohistochemistry (IHC), which detects cellular markers and phenotypes specific to certain diseases through staining with highly specific antibodies [20]. Since IHC can be done on both fresh and formalin-fixed tissues, it serves as a convenient, simple, and cost-effective platform for clinical diagnostics [21]. However, there are several limitations. Although it is not difficult to obtain commercially produced antibodies, there are no standard guidelines for the validation of these antibodies. The interpretation of the slides by pathologists can vary considerably and is affected by how the tissue was processed, stored, and handled. This is exacerbated by the presence of intratumoral biomarker heterogeneity that can cause discordance in the classification of cancer [22]. Furthermore, the lack of standardized guidelines for quantitative and semiquantitative scoring raises concern, especially when it involves clinical decisions. While the American Society of Clinical Oncology (ASCO) and College of American Pathologists (CAP) issued guidelines for the handling of biopsy tissues of breast cancer patients, guidelines for other specimens were not available [23]. At present, most of the emerging biomarkers for CRC are more sensitive and specific as they interrogate the disease down to the DNA and RNA level [24]. The sub-sections below will discuss examples of common and potential biomarkers in CRC.

### 2.1. Methylated Septin9 (mSEPT9)

Screening programs for CRC are highly effective [25] as most CRCs develop from premalignant lesions such as adenomas [26]. Traditional screening methods such as fecal occult blood test (FOBT) and fecal immunochemical test (FIT) are routinely used to screen CRC, but are generally low in sensitivity and specificity [27]. Currently, the screening of CRC-related biomarkers such as carcinoembryonic antigen [28] and insulin-like growth factor-1 [29] through liquid biopsy is made more convenient, but still lacks specificity. Thus, efforts were made to detect tumor-specific DNA in the form of circulating free DNA (cfDNA). cfDNA is continuously shed by healthy and tumor cells into the bloodstream and can reflect the entire tumor genome. Methylated Septin 9 (mSEPT9) DNA is the only FDA-approved methylated biomarker for the screening of CRC through the detection of cfDNA in the serum of CRC patients [30,31]. Warren et al. evaluated the FDA-approved mSEPT9 kit (Epigenomics AG, Germany) and reported an overall sensitivity and specificity in all CRC stages at 90% and 88%, respectively. In early stages of cancer (I and II), the test was 87% sensitive. False-positive rate was reported at 12%. mSEPT9 can be detected in precancerous lesions such as colorectal adenomas [18], but may not be reliably sensitive [32]. In a small prospective study, mSEPT9 was detected in 12% of adenomas with a false-positive rate of 3% [18]. From the year 2012 onwards, more studies were pointing toward the reliability of detecting mSEPT9 for CRC screening, with sensitivities ranging from 73% to 95.6% [33,34,35,36,37]. The heterogeneity of the sensitivities is likely affected by non-neoplastic factors including, but not limited to, demographics, methodology, lifestyle, and sex [37]. Asides from CRC, SEPT9 may also be involved in the tumor progression of leukemia [38], breast [39], ovarian [40], urologic [41], and brain cancers [42]. Nevertheless, the detection of mSEPT9 as cfDNA for CRC has been proven to be effective and should be considered to be included as a routine screening test for CRC.

The role of mSEPT9 in the screening of CRC is well established, but its prognostic role remains unclear. A recent study by Ma and co-workers linked higher levels of mSEPT9 in post-surgery CRC patients to higher cancer recurrence [43]. If validated by future studies, the same FDA-approved kit can be conveniently used in both screening and prognostic tests without extensive optimizations. Similar to mSEPT9, hypermethylations of other DNA biomarkers are also being investigated for their potential role in CRC screening [44]. Methylated DNA are ideal biomarkers for screening as epigenetic alterations occur very early in polyp-to-cancer progression [45]. In addition, there is a plethora of other potential CRC biomarkers in the form of cfDNA that are being studied [46], but will not be reviewed due to space constraints.

### 2.2. Microsatellite Instability (MSI)

Microsatellite instability (MSI) is described as having genes with altered lengths due to small insertions and deletions of short, repetitive DNA sequences throughout the genome [47,48]. These mutations are normally found in the coding single nucleotide repeats of tumor suppressor genes such as activin receptor type 2 and transforming growth factor βR2 [49]. Approximately 15% of all CRC cases are MSI, and patients with MSI-high (MSI-H) have a better prognosis in terms of longer overall survival (OS) and lower metastatic rate, but the tumors are more resistant to adjuvant chemotherapy such as 5-fluorouracil (5-FU) [50]. While MSI-H patients have been shown to be unresponsive to 5-FU, a meta-analysis by Jover et al. found that DNA mismatch repair (MMR) mutational status should not be indicative of chemotherapy efficacy [51]. MSI status by itself is also predictive for treatment response to the anti-programmed cell death 1 (PD-1) checkpoint inhibitor pembrolizumab in metastatic CRC (mCRC) patients. Pembrolizumab is only effective in mCRC patients with MSI-H status [52,53]. The exact interactions and mechanisms between checkpoint inhibitors and MSI status are currently unknown, but its approval by the FDA for the treatment of MSI-H solid tumors is based on several successes in clinical trials, as summarized by Marcus et al. The approval of pembrolizumab marks the first time in history a drug was approved solely based on a common molecular marker instead of the primary site of origin [7]. Recently, however, tumor mutational burden (TMB) is seen as a superior predictive marker over MSI status. TMB is measured in number of mutations/megabase (mut/MB). This was shown in a study by which anti-PD-1 immunotherapy was effective in microsatellite stable (MSS) CRC tumors with high TMB (≥10 mut/MB) [54]. Contrary to other studies demonstrating the lack of efficacy of anti-PD-1 therapy in MSS patients, the importance of TMB over MSI status needs to be investigated further.

### 2.3. KRAS (Kirsten Rat Sarcoma Viral Oncogene Homolog)

The significance of (*KRAS) Kirsten rat sarcoma viral oncogene homolog* mutational profile as a negative predictive biomarker in the treatment response of mCRC using monoclonal antibody (mAb) against epidermal growth factor receptor (EGFR) is well established [55,56,57]. Clinical trials such as PRIME (panitumumab) and CRYSTAL (cetuximab) demonstrated positive response towards anti-EGFR mAb therapies only in wild-type (WT) *KRAS* mCRC patients. This is because *KRAS* activation in *KRAS*-mutated mCRC patients is independent of upstream EGFR activation [58,59]. A subsequent retrospective study by the FDA on seven randomized trials resulted in the approval of cetuximab and panitumumab only in WT *KRAS* mCRC patients [3,10]. Previously, *KRAS* mutation was identified only by mutations in Codon 12 and 13 of Exon 2, which was subsequently found to be insufficient for an accurate prediction of treatment response [60]. Thus, the CRC clinical guideline urges for extended *RAS* mutation testing including *KRAS* and *(NRAS) neuroblastoma RAS viral oncogene homolog* in exon 2 (codons 12 and 13), exon 3 (codon 59 and 61), and exon 4 (codon 117 and 146) [19].

### 2.4. BRAF (v-raf Murine Sarcoma Viral Oncogene Homolog B1)

*BRAF (v-raf murine sarcoma viral oncogene homolog B1)* mutation occurs in 10% of CRC cases, with most of the mutations being presented in Codon 600 [61]. Recent evidences suggest that *BRAF* mutation is a better predictor for the determination of anti-EGFR therapy responses than *RAS* status. This is exemplified by the lower overall response rate (ORR) of anti-EGFR mAb in mutant *BRAF* compared with mutant Exon 2 *KRAS* [62]. Additionally, *BRAF* mutation is associated with the promoter methylation of an MMR gene, MLH1 (human mutL homolog 1), where a positive *BRAF* mutation is normally accompanied with negative MMR mutation status. The negative mutation status of MMR is important for the prediction of MSI status [63,64]. In essence, patients with *BRAF* mutations are normally MSS, and are thus less likely to benefit from pembrolizumab treatment. *BRAF* mutation may also indicate poor prognosis in CRC patients [65], but is only demonstrated in *BRAF*-mutated patients with MSS status, as reported by Roth et al. The study showed that *BRAF* mutations hold no prognostic significance in patients with MSI-H [66]. In contrast, a recent meta-analysis of 1164 nonmetastatic CRC patients with MSI-H showed that *BRAF*V600E mutation does indicate poor prognosis in terms of OS [67]. Whilst the mutational testing of *BRAF* is recommended in the CRC clinical guidelines for prognostic stratification, and MMR status identification, findings suggest that *BRAF* mutation alone is insufficient for a full diagnosis of CRC [19].

### 2.5. Other Potential Biomarkers

The complex genetic nature and heterogeneity of CRC necessitate the detection of a combination of biomarkers for a more accurate diagnosis. Thus, efforts are continuously made to validate additional CRC biomarkers. The sub-sections below will review CRC biomarkers that may potentially be incorporated into routine clinical diagnostics.

#### 2.5.1. Programmed Death-Ligand 1 (PD-L1)

Programmed Death-Ligand 1 (PD-L1) expression is potentially predictive for the treatment response of pembrolizumab since high PD-L1 expression has been associated with MSI-H status [68,69]. The association between high PD-L1 expression and MSI-H status has, however, been contrasted in another study involving a larger cohort of almost 1,500 samples [70]. It is speculated that these large variations could be attributed to the difference in immunohistochemistry staining methods and scoring criteria due to the spatial and temporal heterogeneity of PD-L1 expression in mCRC patients [71]. Another study concluded that the effectiveness of the checkpoint inhibitor appears to be independent of PD-L1 expression level by tumor cells [72]. Collectively, it is evident that these limitations will need to be addressed before any clinical applications involving PD-L1 expression can be applied on CRC patients.

#### 2.5.2. Phosphatidylinositol-4,5-bisphosphate 3-kinase, Catalytic Subunit Alpha (PIK3CA)

*PIK3CA* mutation has been studied for CRC treatment [73]. It is indicated that the PIK3CA exon 20 mutation confers resistance against anti-EGFR mAb therapy in CRC patients. The response rate (RR) was reported to be as low as 0%, together with shorter progression-free survival (PFS) [74]. Conversely, another study demonstrated that PIK3CA did not significantly affect resistance against cetuximab [75]. An independent lab-developed test detecting *H1047R* and *E545K* mutations showed sensitivities of 5% and 10% mutant allele fractions, respectively [76].

#### 2.5.3. Phosphatase and Tensin Homolog (PTEN)

Another biomarker proposed to have predictive and prognostic potential in CRC treatment is *Phosphatase and Tensin Homolog (PTEN)* [77]. A study with 67 CRC patients demonstrated that 100% of the patients with negative expression of PTEN exhibited disease progression following treatment with cetuximab, whereas 30% of the PTEN expression patients showed reduced disease progression [78]. Nevertheless, a study of a larger cohort found that it was not associated to the RR [79]. Another study showed that the negative expression of PTEN only negatively correlates to cetuximab response in tumor metastases but not primary tumor of CRC [80]. Interestingly, some studies showed promising results where PTEN gene alterations resulted in largely poor OS [81,82], while other findings showed no correlation between patients’ survival with changes in PTEN expression [83,84]. Together, the credibility of PTEN as a potential predictive and prognostic marker cannot be determined as of current, and further investigations need to be carried out.

#### 2.5.4. Human Epidermal Growth Factor Receptor 2 (HER2)

Human Epidermal Growth Factor Receptor 2 (HER2) is a proto-oncogene in the EGFR family that is mostly known for its oncogenic role in breast cancer [85]. In rare cases, HER2 is amplified in approximately 5% of mCRC patients with WT *KRAS* exon 2. This gene exhibits a high concordance rate in both primary and metastatic tumors of CRC [86] and is mutually exclusive with mutations to *NRAS*, *KRAS*, and *BRAF* [87]. The amplification of HER2 in relation to anti-HER2 treatment response in mCRC patients was evaluated by the phase II HERACLES trial combining two anti-HER2 drugs, trastuzumab and lapatinib. The results of the trial suggested that HER2 amplification may have a potential role as a clinical predictive marker for CRC, but the extreme rarity of this genetic alteration proves challenging for the validation of these results in a phase III clinical trial [88].

#### 2.5.5. Micro-RNA (miRNA)

MiRNAs as biomarkers have received increasing attention recently for their predictive, diagnostic, and prognostic roles in CRC [89]. There are several miRNAs that are of importance to CRC. MiR-135a and miR-135b, for example, contribute to the regulation of the Wnt/Wingless pathway through the downregulation of APC (Adenomatous polyposis coli) gene [90] and can potentially act as prognostic markers [91,92]. Additionally, miR-17-3p and miR-92a (pooled sensitivity, 76%, specificity, 64%) can upregulate the cell proliferation of colon cancer cells and have been found to be elevated in the plasma and CRC tissues [93,94,95]. MiR-211, on the other hand, can act as a marker for both the diagnosis and prognosis of CRC [96], while miR-133a can be potentially used in predicting response to EGFR inhibitors [97].

#### 2.5.6. Consensus Molecular Subtypes (CMS)

**Figure 2:**
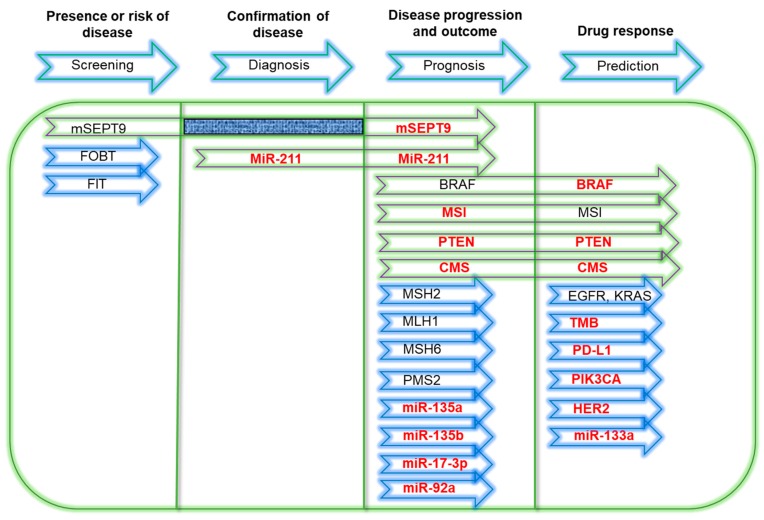
Simplified summary of CRC biomarkers. Green arrows represent biomarkers that have more than one role. Biomarkers colored in red are potential CRC biomarkers that require additional validations. BRAF- v-raf murine sarcoma viral oncogene homolog B1; FIT- fecal immunochemical test; CMS- consensus molecular subtypes; EGFR- epidermal growth factor receptor; FOBT- fecal occult blood test; HER2- human epidermal growth factor receptor 2; KRAS- Kirsten rat sarcoma viral oncogene homolog; miR- microRNA; MLH1- human mutL homolog 1; mSEPT9- methylated Septin 9; MSH2- human mutS homolog 2; MSI- Microsatellite instability; PD-L1- Programmed death-ligand 1; PIK3CA- phosphatidylinositol-4,5-bisphosphate 3-kinase, catalytic subunit alpha; PMS2- PMS1 homolog 2, mismatch repair system component; PTEN- Phosphatase and tensin homolog; TMB- tumor mutational burden.

Recently, the possibility for the consensus molecular subtypes (CMS) of CRC to be used as biomarkers was explored. The subtypes are grouped into four based on the tumor gene expression pattern and are described as: (1) CMS1—strong immune activation, hypermutated, and MSI-H; (2) CMS2—epithelial and canonical with marked WNT and MYC signaling; (3) CMS3—epithelial with evident metabolic dysregulation; and (4) CMS4—mesenchymal with stromal invasion, angiogenesis and prominent transforming growth factor-β activation [98,99]. Prognostic and predictive values of CMS in mCRC patients independent of cancer staging were evaluated in multiple retrospective analysis. Collectively, CMS was shown to be significantly associated to lower OS and PFS. In terms of OS, CMS1 was associated to worst outcome in contrast to CMS2. Notably, positive drug response to bevacizumab and cetuximab was demonstrated specifically in CMS1 and CMS4, respectively. [100,101,102,103]. Significant correlations with known CRC biomarkers such as MSI-H, *KRAS,* and *BRAF* mutations were also reported [100]. A simplified summary of the biomarkers reviewed in Section 2.1, Section 2.2, Section 2.3 and Section 2.4 is presented in Figure 2 [18,19,43,54,56,57,70,74,78,79,88,91,92,93,94,96,97,100,101,102,103,104,105].

## 3. Current Molecular Diagnostic Technologies

With advancements in technology, major progress has been made in understanding how variations in the genetic alterations of CRC can contribute toward clinical management. These advances have led to the development of precision medicine, which offers individualized medical care based on the patient’s personal information and unique genetic profile. This concept has been proven to be successful in improving clinical outcomes compared with the relatively conventional method of indiscriminate radio/chemotherapy [106]. In fact, the list of drugs targeting specific genetic alterations approved by the FDA is rapidly growing for the treatment of advanced-stage solid tumors. Hence, it is important to understand and appreciate current and developing molecular methods available to diagnose CRC and the variety of molecular technologies available to map out the individual’s unique molecular profile for efficient treatment strategies.

Before polymerase chain reaction (PCR) was invented, the detection of nucleic acid biomarkers was challenging due to scarcity of nucleic acid and tissue heterogeneity in samples. Now, tumor-specific DNA can be amplified to detectable levels through the more sensitive quantitative PCR (qPCR) [107], while RNA-based biomarkers such as miRNA can be detected using quantitative reverse transcription PCR (RT-qPCR) [94]. As technology improves, extremely scarce biomarkers such as tumor-specific cfDNA can be detected using qPCR, digital PCR, and NGS. Digital PCR is the latest variation of PCR that has since entered the diagnostic market recently, when detection of very rare events or gene variants in patient sample is challenging using qPCR. NGS, on the other hand, refers to high-throughput nucleic acid sequencing platforms using fragmented and PCR-amplified DNA, with performance comparable to digital PCR in terms of rare event detection [108]. The sub-sections below will discuss updates on the use of current molecular diagnostic technologies to detect major CRC-related biomarkers such as *KRAS, BRAF, MSI,* and so on.

### 3.1. Detection of KRAS and BRAF Mutations

For CRC, the mutational status of the genes *KRAS* and *BRAF* receives additional attention as they can negatively affect the patient’s response towards anti-EGFR therapies, with the prevalence of somatic *KRAS* mutations being 40% [109]. A myriad of molecular assays can detect these mutations with limit of detections ranging from 10%–20% mutant allele for Sanger sequencing [110], around 5% for pyrosequencing [110,111] and high-resolution melt (HRM) curve analysis [110,112], to 1–5% for qPCR assays [113,114]. While direct sequencing remains the gold standard for *KRAS* and *BRAF* mutations detection, the method is not popular as it is laborious and lacks sensitivity [106,115].

#### 3.1.1. PCR-Based Detection

Currently, there are several FDA-approved assays for the detection of *KRAS* and *BRAF* mutations, including cobas 4800 *BRAF* V600 mutation assay [116], cobas 4800 *KRAS* mutation assay, and therascreen *KRAS* assay [117,118]. These assays are FDA-approved for specific samples, but they are not suitable for use in specimens containing limited tissue such as fine needle aspiration and core biopsy [106]. A study by Nordgård et al. compared the performance and cost-effectiveness of two relatively new sensitive molecular methods for the detection of *KRAS* mutations called amplification refractory mutation system (ARMS) PCR assay and peptic nucleic acid (PNA) clamp PCR [119]. The ARMS PCR assay is an allele-specific PCR that detects a specific known mutated form of a gene [47], while the PNA clamp PCR is capable of selective amplification of nucleic acid sequences that differ by a single base [120]. In comparison, the PNA clamping assay was in favor as it is approximately 20 times cheaper than ARMS and demonstrated higher sensitivity levels [119]. Another method that utilizes a modified PCR protocol termed as coamplification at lower denaturation temperature PCR (COLD-PCR) was designed to detect *KRAS* and *BRAF* V600E mutations in combination with HRM. Compared with traditional PCR and direct sequencing that picked up 57 *KRAS*/*BRAF* mutations out of 117 CRC samples, this method detected 72 *KRAS*/*BRAF* mutations, which translates to a 26.3% increase in detection. In addition, this method does not require time-consuming procedures and expensive equipment. Thus, it can be considered for diagnostic uses [121].

In respect of technology, many of the available routine molecular tests are heading toward automation, including EGFR mutation detection. The Idylla (Biocartis, Belgium) is a CE-IVD (CE Marking *In Vitro* Diagnostic for Medical Devices) marked fully-automated qPCR diagnostic platform for EGFR mutations that is simple to perform (hands-on time of less than 2 min), does not require molecular expertise to run, is reliable, rapid, and has a small footprint [122]. Its *KRAS* mutation test allows the simultaneous detection of 21 mutations in clinically relevant codons (12, 13, 59, 61, 117, and 146) from FFPE (formalin-fixed paraffin-embedded) tissue samples, while its *NRAS*–*BRAF* mutation test can detect codons 12, 13, 59, 61, and 117 for *NRAS* and V600 mutations for *BRAF* [123]. Its ease of use, lack of expertise required to operate the system, and rapid turn-around time allows for the system to be placed in most clinical settings.

#### 3.1.2. Nucleic Acid Hybridization Assay

There are cost-effective alternatives for the detection of *KRAS* and *BRAF* other than qPCR. This includes single nucleotide primer extension (SNaPshot) assay and reverse hybridization StripAssay. The SNaPshot is a flexible lab-developed test, while the StripAssay is a commercially available test [115,124,125]. For *KRAS*, the detection limits when compared with direct sequencing are 10% and 1%, respectively [115]. In terms of sensitivity and turn-around time, the *KRAS* StripAssay is more favorable as it is a rapid and sensitive test that can be utilized when few tumor cells are present. For *BRAF*, Magnin et al. demonstrated similar results among SNaPshot, direct sequencing, and HRM [125]. Evaluation data on the performance of *BRAF* using the StripAssay is, however, lacking and warrants further investigation. In short, SNaPshot and the *KRAS* StripAssay are suitable for laboratories lacking dedicated equipment, but Sarasqueta et al. has warned for *KRAS*-mutant to be confirmed to reduce the risk of false positives [115].

#### 3.1.3. Pre-PCR Isolation of Circulating Tumor Cells (CTCs)

Since it is difficult to obtain sufficient or appropriate tumor samples for *KRAS* genotyping, an alternative method is required. Thus, circulating tumor cells (CTCs) have garnered a strong reaction from researchers and clinicians as they are thought to represent the tumor in real-time. The extremely low levels of DNA in CTCs can be detected through qPCR with HRM or droplet digital PCR (ddPCR). When compared, ddPCR was able to detect less than one *KRAS* mutant cell per mL of neat blood (0.05% mutant ratio) while qPCR (TaqMeltPCR) and HRM could only detect up to 0.5% mutant ratio. In addition to its high sensitivity, the noninvasive nature of this procedure also allows for future opportunities to screen, diagnose, and monitor treatment or relapse in real-time without much discomfort to the patients [126]. One of the biggest hurdles to downstream analysis of CTCs is the effective and reliable method of isolating CTCs. There are several commercially available equipment for the isolation of CTCs through physical and biochemical properties such as size exclusion, deformability, and surface marker expression, but they are not without challenges. The size, rigidity, and surface marker expression pattern of CTCs are known to be highly variable. Furthermore, marker-based isolation of CTCs is only possible on the availability of the particular antibody [127].

#### 3.1.4. Circulating Free DNA (cfDNA) Detection

Moving forward, *RAS* mutations can now be detected as cfDNA without the need to isolate CTCs. In a study published in 2018, researchers compared three diagnostic platforms for *RAS* mutations detection through cfDNA: ddPCR (Bio-Rad), NGS (Illumina), and BEAMing/OncoBEAM-RAS-CRC (BEAM, Sysmex Inostics). Tissue biopsies and time-matched blood samples were collected for the simultaneous comparison of mutation profiles between cfDNA and FFPE. The digital PCR-based BEAM reached a detection threshold of 0.03%, significantly lower than the detection thresholds of the two compared platforms at 0.5–1%. The superior sensitivity of the BEAM allowed the detection of *KRAS* mutations in 5/19 FFPE profiles shown to be negative. Based on the comparison of sensitivity, specificity, positive predictive value (PPV), and negative predictive value (NPV) among the three platforms (Table 1) [108], the BEAM is superior in most aspects, except for the specificity, which is slightly lower than that of the other two platforms. While the NGS is more suitable for the detection of a large coverage of mutations, the results are oftentimes confirmed with ddPCR [108]. In clinical settings where there is a need to balance the coverage of large mutation profiles with specificity of a particular mutation, it will be useful to have the BEAM together with the NGS as a complementary platform. As shown in Table 1, NGS may be a better choice, but the decision on which platform to select will require the consideration of footprint, cost, and turn-around-time (TAT). Furthermore, the clinical relevance of detecting mutations at very low allelic frequency was not assessed. While the results of ddPCR are less informative, it has the shortest TAT of only 8 h for the complete mutational analysis of *KRAS* exon 2. The more extensive mutational analysis of *KRAS* from BEAM and NGS will require around 2 days and 7 days, respectively. In terms of costing, NGS and the BEAM are comparable but cost twice as much compared with the ddPCR [108].

### 3.2. Detection of Microsatellite Instability (MSI)/Microsatellite Stable (MSS) Status

Similar to the detection of other biomarkers, MSI can be detected through PCR-based methods and NGS. The versatility of NGS allows for the simultaneous detection of thousands of different microsatellite loci with a limit of detection of 1% MSI in MSS background. However, the use of NGS for MSI detection is uncommon, as data interpretation requires specific algorithms and computational methods [128]. Notably, MSI can also be detected indirectly through the detection of miRNA biomarkers.

There are several methods in which MSI can be detected. Capillary electrophoresis fragment analysis is one of the earliest molecular method using PCR-amplified samples, but presents with a low limit of detection of 1% to 10% [128]. This method usually interrogates epiphenomenon related to MSI, such as MMR gene loci [129]. The 1997 National Cancer Institute-sponsored MSI workshop recommends the interrogation of five microsatellite loci; three dinucleotide (D2S123, D5S346, and D17S250) and two mononucleotide repeats (BAT-25, BAT-26), which led to the development of the Bethesda panel. This assay amplifies the repeats through a single multiplex PCR reaction, followed by analysis using capillary electrophoresis. MSI-H is reported when two or more loci are instable, while instability at a single locus or no instability will be reported as MSI-low and MSS, respectively. Commercially, Promega Corp developed the MSI Analysis System version 1.1 that detects five mononucleotide (BAT-25, BAT-26, NR-21, NR-24, and MONO-27) and two pentanucleotide repeats (PentaC and PentaD). Like the previous assay, capillary electrophoresis is subsequently used for analysis. MSI-H is reported when two or more mononucleotide loci are instable, while instability at a single mononucleotide locus represents MSI-low, and no instability represents MSS. Murphy et al. compared the two assays with 34 CRC patient samples and found that the concordance rate was at 85%. MSI-H and MSS cases were fully concordant on both assays. While both assays performed adequately in detecting MSI cases, the authors concluded that the MSI Analysis System was superior and will be a better option to resolve cases of MSI-low into either MSS or MSI-H [130]. In another study, the Promega MSI Analysis System version 1.2 was compared to a new “one mononucleotide” MSI marker test, which detects T25 mononucleotide repeat of the caspase 2 gene (*CAT25*). The CAT25 MSI test claims to be a simplified assay with reduced cost and may improve efficiency. Based on the results, the authors concluded that the CAT25 assay performed equally well to the commercial kit and should be considered as a replacement to a five markers test, or at least, be included in the MSI detection panel [131]. Additionally, an MSI gene expression assay with 64 genes has been made available as a diagnostic assay, which helps to accurately identify MSI-positive and MSI-like patients that are not easily recognized by IHC or PCR. Furthermore, this assay does not require a reference paracancerous tissue from the patient for comparison [65].

### 3.3. miRNA Detection

MSI status can also be tested through the detection of miRNA biomarkers in the plasma as cell-free form. One of the methods is through RT-qPCR, which measures the quantity of miRNA indirectly in real-time [132]. Microarray, which utilizes in situ hybridization of predesigned probes to the target miRNA is also a reliable method to quantify the amount of miRNA in the sample [133]. An MSI oligonucleotide microarray diagnostic assay based on spotted locked nucleic acid (LNA) can be used to profile the expression of up to 315 miRNAs [89]. While RT-PCR and microarray are highly sensitive and specific, the operational cost is expensive. Dedicated equipment, facilities, and trained personnel are also required to run these tests [134,135]. Thus, these methods may not be suitable for clinics or settings with poor resources.

Alternatively, resource-lacking clinical settings may opt for lateral flow nucleic acid strip assay using gold nanoparticles [135]. This assay has been demonstrated to be more apt as a point-of-care test as it is sensitive, inexpensive, rapid, and simple to use [135,136]. Similar to the microarray system, this assay utilizes predesigned probes conjugated to gold nanoparticles targeting specific miRNAs, leading to the formation of visible bands on the strip [47]. The results are semiquantitative as the intensity of the band increases with the quantity of the miRNA, but can be made more quantitative with the use of a quantitative detection platform [135,136]. It was shown that miRNAs can be quantified to as low as 1 fmol, and with the use of silver enhancement, it can be as low as 5 amol [134].

### 3.4. Detection of Other Biomarkers

In theory, most of the molecular biomarkers discussed other than *KRAS*, *BRAF*, and MSI can be detected using similar diagnostic platforms such as qPCR and NGS. This is shown in several studies attempting to validate the use of additional biomarkers for CRC diagnostics. A brief summary of CRC biomarkers and their respective diagnostic platforms from Section 2 and 3 is tabulated in Table 2.

## 4. Challenges and Limitations

### 4.1. Choice of Molecular Platform

For cancer diagnosis and screening, accuracy, specificity, and fast TAT should be given critical attention [137]. qPCR is currently the choice of molecular method for biomarker detection as many tests are validated and it is more readily available than digital PCR and NGS. In addition, test results can be available within a day. However, the throughput is limited to several targets, and prior knowledge of the target DNA is required. Conversely, a single run of NGS is capable of providing invaluable information including mutations, chromosomal rearrangements, and copy number alterations without any prior knowledge of the targets [138], although it may take up to 7 days for results to be available [108]. Superior sensitivity, specificity, and throughput must not be the sole reason for the molecular platform chosen. Instead, consideration must be given to the type of clinical application, cost effectiveness, and the necessity for superior performance, as it is not possible for all medical settings to incorporate advanced molecular laboratories. A centralized and highly specialized cancer diagnostic lab will need to be established in major cities or states to process samples from surrounding clinical settings, but may have several drawbacks. The need to handle sensitive clinical samples during transportation and process samples in batches will lead to a longer TAT. Consequently, some critically ill patients may have to wait for several days or up to weeks before receiving appropriate treatment [19,139].

### 4.2. Laboratory Operations

Guidelines for CRC molecular testing have been established in a joint effort by the American Society for Clinical Pathology (ASCP), American Society of Clinical Oncology (ASCO), Association for Molecular Pathology (AMP), and the College of American Pathologists (CAP). For clinical sample management and laboratory operations, the recommendations by the guidelines will be summarized and discussed here. Firstly, policies should be established in all laboratories to ensure efficient allocation of tissue for molecular tests, especially in scarce specimens. Pathologists can contribute to this by evaluating tissue quantity, quality, and malignant tumor cell fraction. Another way is to limit the number of tissue fragments per individual cassette. Prior assessment of the patients’ previous CRC status will contribute to unnecessary repeat tests. The usage of appropriate tissue type may also aid in limiting the wastage of precious tissue; the guidelines recommend using recurrent or mCRC tissues for predictive biomarker testing involved in cancer treatment given that samples are available and adequate, followed by primary tumor tissue. For molecular biomarker mutational testing, FFPE tissue samples are considered acceptable. However, an alteration to the tissue processing protocols or the use of other specimens (including samples of cytologic origins) necessitates additional validation [19].

Another recommended practice to consider is to provide clinically appropriate turn-around time (TAT), which will contribute to the alleviation of patient anxiety and the execution of timely patient management decisions. This can be achieved through the optimal utilization of relevant molecular and IHC tests, possibly through multiplexed assays. In general, nonacute biomarker test results should be available within 10 days of test ordering [19,140]. The 10 day timeframe should include the time taken for test orders to reach the laboratory, tissue block retrieval, and shipment of said blocks to the performing laboratory. Every effort should be taken by the laboratory to minimize delays in tissue block retrieval, processing time, and the availability of results [19].

## 5. Conclusions and Future Perspectives

It is important to understand the epidemiology, etiology, mechanisms, and how to improve diagnostics and treatment in CRC. Owing to the continuous research of CRC by dedicated researchers, the mechanisms underlying CRC are now more understood, resulting in extensive diagnostic and treatment options. Detection of circulating free DNA from liquid biopsy is currently in trend as it is less invasive and extremely sensitive. Notably, the detection of methylated SEPT9 in the plasma of CRC patients has been shown to be a reliable screening tool and has been FDA-approved as the first liquid biopsy screening tool for CRC. These sensitive diagnostic tools will shape how and what treatments are prescribed, as certain targeted treatments are only effective in certain genotypes of CRC. Evidently, there will be limitations to the implementation of such tests and treatments in all healthcare settings. Furthermore, as with all new technology, validation studies and standardization of operations and methodologies remain a challenge. In essence, CRC continues to be one of the most prevalent cancers in the world and is mostly detected in advanced stages. Thus, current and future research should strongly emphasize on early screening of CRC as it is one of the most effective way to prevent CRC. The future of CRC prevention, diagnostic, and treatment lies in the decentralization of molecular tests. Currently, extremely sensitive molecular methods for biomarker detection require sophisticated equipment, facilities, and trained personnel, resulting in the need for centralization and longer TAT. This necessitates the development of simple, automated, and robust biomarker detection platforms with smaller footprints.

## Figures and Tables

**Table 1:** Comparison of the sensitivity, specificity, PPV (positive predictive value), and NPV (negative predictive value) of ddPCR, BEAM, and NGS for *KRAS* detection [108].

Platform	Sensitivity (%)	Specificity	PPV (%)	NPV (%)
ddPCR	47	77	70	55
BEAM	93	69	78	90
NGS	73	77	79	71

**Table 2:** Summary of CRC biomarkers and their respective diagnostic platforms reviewed.

Biomarker	Diagnostic Platform	References
mSEPT9	• Methylation-specific real-time PCR	[18]
MSI	• Immunohistochemistry	[64]
	• Next-generation sequencing (NGS)	[89]
	• Fragment analysis	[128]
	• Gene expression assay	[130]
	• miRNA microarray	[89]
TMB	• Whole exome sequencing	[54]
*KRAS*	• qPCR (Roche Cobas 4800 *KRAS* mutation, Qiagen therascreen *KRAS*, Biocartis *KRAS* mutation assay)	[117,122]
	• Direct sequencing	[106]
	• Pyrosequencing	[111]
	• Next-generation sequencing (NGS)	[108]
	• High-resolution melt curve (HRM)	[110]
	• Amplification refractory mutation system (ARMS) PCR	[119]
	• Peptic nucleic acid (PNA) clamp PCR	[119]
	• COLD-PCR	[121]
	• Single nucleotide primer extension (SNaPshot)	[115]
	• Reverse hybridization *KRAS* StripAssay	[115]
	• Digital PCR (Bio-Rad droplet digital PCR, Sysmex BEAMing)	[108]
*BRAF*	• qPCR (Roche Cobas 4800 *BRAF* V600, Biocartis Idylla *NRAS-BRAF* mutation assay)	[117,122]
	• Direct sequencing	[106]
	• Pyrosequencing	[111]
	• Next-generation sequencing (NGS)	[108]
	• High-resolution melt curve (HRM)	[110]
	• COLD-PCR	[121]
	• Immunohistochemistry	[106]
	• Single nucleotide primer extension (SNaPshot)	[125]
	• Reverse hybridization *BRAF* StripAssay	[124]
miRNA	• Microarray—spotted locked nucleic acid (LNA)	[89]
	• RT-qPCR	[132]
	• Lateral flow nucleic acid strip assay using gold nanoparticles	[47]
PD-L1	• Immunohistochemistry	[68]
PIK3CA	• Real-time PCR	[76]
	• Immunohistochemistry	[73]
	• Gene sequencing	[73]
HER2	• Immunohistochemistry	[86]
	• Quantitative reverse transcription PCR (RT-qPCR)	[85]
PTEN	• Indirect immunofluorescence	[78]
	• Immunohistochemistry	[77]
CMS	• Gene expression microarray	[99]

## Abbreviations

APC	Adenomatous polyposis coli
ARMS	Amplification refractory mutation system
*BRAF*	B-Raf protein
cfDNA	Circulating free DNA
COLD-PCR	Coamplification at lower denaturation temperature PCR
CRC	Colorectal cancer
CTC	Circulating tumor cells
ddPCR	Droplet digital PCR
DNA	Deoxyribonucleic acid
EGFR	Epidermal growth factor receptor
FDA	Food and Drug Administration
FFPE	Formalin-fixed paraffin embedded
FIT	Fecal immunochemical test
FOBT	Fecal occult blood test
HER2	Human epidermal growth factor 2
IHC	Immunohistochemistry
*KRAS*	Kirten rat sarcoma viral oncogene homolog
LNA	Locked nucleic acid
mAb	monoclonal antibody
mCRC	metastatic colorectal cancer
miRNA	microRNA
MMR	Mismatch repair
MSI	Microsatellite instability
NGS	Next-generation sequencing
NRAS	Neuroblastoma RAS viral oncogene homolog
ORR	Overall response rate
OS	Overall survival
PD-L1	Programmed cell death protein ligand 1
PFS	Progression-free survival
PNA	Peptide nucleic acid
RR	Response rate
RT-qPCR	Quantitative reverse transcription PCR
SEPT9	Septin 9
TKI	Tyrosine kinase inhibitor
TMBWNT	Tumor mutational burdenWingless and int-1
WT	Wild-type
5-FU	5-fluorouracil
